# The Chemical Environment at Maturation Stage in *Pinus* spp. Somatic Embryogenesis: Implications in the Polyamine Profile of Somatic Embryos and Morphological Characteristics of the Developed Plantlets

**DOI:** 10.3389/fpls.2021.771464

**Published:** 2021-11-26

**Authors:** Antonia Maiara Marques do Nascimento, Luiza Giacomolli Polesi, Franklin Panato Back, Neusa Steiner, Miguel Pedro Guerra, Ander Castander-Olarieta, Paloma Moncaleán, Itziar Aurora Montalbán

**Affiliations:** ^1^Neiker-BRTA, Centro de Arkaute, Campus Agroalimentario de Arkaute, Arkaute, Spain; ^2^Laboratório de Fisiologia do Desenvolvimento e Genética Vegetal, Universidade Federal de Santa Catarina, Florianópolis, Brazil; ^3^Departamento de Botânica, Universidade Federal de Santa Catarina, Florianópolis, Brazil

**Keywords:** Pinus halepensis, Pinus radiata, sugars, amino acids, osmolality

## Abstract

Changes in the chemical environment at the maturation stage in *Pinus* spp. somatic embryogenesis will be a determinant factor in the conversion of somatic embryos to plantlets. Furthermore, the study of biochemical and morphological aspects of the somatic embryos could enable the improvement of somatic embryogenesis in *Pinus* spp. In the present work, the influence of different amino acid combinations, carbohydrate sources, and concentrations at the maturation stage of *Pinus radiata* D. Don and *Pinus halepensis* Mill. was analyzed. In *P. radiata*, the maturation medium supplemented with 175 mM of sucrose and an increase in the amino acid mixture (1,100 mgL^–1^ of L-glutamine, 1,050 mgL^–1^ of L-asparagine, 350 mgL^–1^ of L-arginine, and 35 mgL^–1^ of L-proline) promoted bigger embryos, with a larger stem diameter and an increase in the number of roots in the germinated somatic embryos, improving the acclimatization success of this species. In *P. halepensis*, the maturation medium supplemented with 175 mM of maltose improved the germination of somatic embryos. The increase in the amount of amino acids in the maturation medium increased the levels of putrescine in the germinated somatic embryos of *P. halepensis*. We detected significant differences in the amounts of polyamines between somatic plantlets of *P. radiata* and *P. halepensis*; putrescine was less abundant in both species. For the first time, in *P. radiata* and *P. halepensis* somatic embryogenesis, we detected the presence of cadaverine, and its concentration changed according to the species.

## Introduction

Somatic embryogenesis has been suggested as the most promising method for vegetative propagation for a large number of conifers (Gupta et al., 1993), and it can help to capture the greatest benefits from traditional breeding programs by multiplying trees with desirable characteristics for plantation forestry (Pullman and Bucalo, 2014).

*Pinus radiata* D. Don is one of the most planted pine species in the world, and it is widely used to produce quality wood (Escandon et al., 2017; Fuentes-Sepulveda et al., 2020). Alternatively, *Pinus halepensis* Mill. is a species with high forest importance for reforestation because it can survive under low precipitation levels, and it has higher tolerance to thermal and drought stress (Gazol et al., 2017; Manrique-Alba et al., 2020; Quinto et al., 2021).

Previous studies carried out in our laboratory have demonstrated that *P. radiata* and *P. halepensis* showed different responses to abiotic stress such as drought and high temperature (do Nascimento et al., 2020). Also, in previous experiments, we have focused on the optimization of somatic embryogenesis (SE) processes. In this sense, the optimization of initiation and proliferation stages of SE in *P. radiata* (Montalbán et al., 2010, 2012) and *P. halepensis* (Montalbán et al., 2013) has been our objective in recent years. Recently, some studies focused on promoting and improving the embryogenic process in terms of quality and quantity of somatic embryos (ses) obtained by reduction of water availability in *P. radiata* (Garcia-Mendiguren et al., 2016; Castander-Olarieta et al., 2020) and *P. halepensis* (Pereira et al., 2016, 2017; do Nascimento et al., 2020) have been carried out. However, the rate of germination (%) and the subsequent conversion into plantlets is sometimes low (do Nascimento et al., 2020), and additional research is needed to increase somatic plantlet regeneration (Montalbán and Moncaleán, 2019).

The source of organic nitrogen is an important component of the culture media for the success of the SE process, and in this sense, amino acids are commonly used to improve the maturation of ses, modifying its type and concentration (Gerdakaneh et al., 2011; Satish et al., 2016; Dahrendorf et al., 2018). As Maruyama et al. (2021) reported, the presence of organic nitrogen improved the production of ses. Casein and glutamine are usually used in the SE of conifers (Afele and Saxena, 1995; Pullman and Bucalo, 2014; Dahrendorf et al., 2018), but, in some species, such as *Pinus patula* Schiede ex Schltdl. and Cham. or *P. radiata*, it has been seen that mixtures of amino acids can be more beneficial for the process (Malabadi and Van Staden, 2005; Montalbán et al., 2010).

The beneficial effect of chemical environment modification in a certain stage of the process can be witnessed not only in that stage but also in the following ones (Dahrendorf et al., 2018; Castander-Olarieta et al., 2019; do Nascimento et al., 2020). In this case, the presence of a carbon source in the culture medium is necessary due to the heterotrophy conditions in which the cells grow (Yaseen et al., 2013). In addition, the carbohydrates used in the SE increase the osmolality of the culture medium (Smeekens et al., 2010; Kube et al., 2014). The most common source of carbohydrates in the culture medium is sucrose, but other carbohydrates such us maltose have been used in other *Pinus* species such as *Pinus nigra* Arn. (Salaj et al., 2019). Therefore, due to the different responses observed in the SE of various species, it is necessary to carry out a study about the effect of different carbohydrates and concentrations (Yang et al., 2020).

Polyamines are considered to be a class of compounds related to cellular proliferation (Bais and Ravishankar, 2002; Jimenez, 2005) and play an important role in the regulation of SE (Silveira et al., 2004; Jin et al., 2020; Sundararajan et al., 2021). Moreover, polyamines (PAs) could be associated with the stress caused in plant cells, protecting them and upregulating stress-related genes (Alcazar et al., 2020; Kielkowska and Adamus, 2021). PAs levels vary according to each stage of SE, for example, during the intense cell division observed in the proliferation phase; higher diamine putrescine (Put) contents were reported (Minocha et al., 2004), whereas, in the maturation stage, where intense cell differentiation occurs, higher levels of triamine spermidine (Spd) and tetraamine spermine (Spm) were observed (Minocha et al., 2004; Gemperlova et al., 2009). In conifers, the accumulation of high levels of PAs was correlated also with the conversion of ses in plants (Gemperlova et al., 2009). Moreover, PAs have been considered as markers of SE competence in several species, such as *Oryza sativa* L. (Shoeb et al., 2001), *Medicago sativa* L. (Huang et al., 2001), and *Panax ginseng* C.A. Meyer (Monteiro et al., 2002).

In plants, the three most common PAs are Put, Spd, Spm (Bouchereau et al., 1999; Minocha et al., 2014), and cadaverine (Cad), which are rarely reported (Jancewicz et al., 2016). Additionally, as the amino acids are precursors of PAs biosynthesis, the exogenous addition of amino acids in the medium culture can directly influence PA profiles (Bagni and Tassoni, 2001; Pullman and Bucalo, 2014). The biosynthesis of Put, Spd, and Spm is directly related to arginine and ornithine (de Oliveira et al., 2018), while the biosynthesis of Cad is associated with lysine and ornithine (Liu et al., 2014). In *Araucaria angustifolia* Bert. O. Ktze., the ratio of PAs determined the transition from embryonal masses (EMs) to ses (Farias-Soares et al., 2014).

The aim of this work was to evaluate the influence of the different amino acid mixtures and carbohydrate sources, applied during the maturation stage of the SE, on the number and morphology of the ses obtained, their PAs profile, and the morphological characteristics of the *P. radiata* and *P. halepensis* plantlets developed.

## Materials and Methods

### Plant Material

Embryonal masses (Ems) were obtained from immature cones of *P. radiata*, collected from four mother trees in a seed orchard set-up by Neiker-BRTA in Deba (Spain; latitude: 43°16′59″N, longitude: 2°17′59″W, elevation: 50 m) and of *P. halepensis*, collected from five mother trees in Berantevilla (Spain; latitude: 42°40′57.14″N, longitude: 2°51′29.95″W, elevation: 473 m). The immature seeds of both species were extracted and surface sterilized, following Montalbán et al. (2010, 2012). Seed coats were removed, and intact megagametophytes were excised out aseptically and cultured on initiation media for both species; for *P. radiata*, the basal medium was Embryo Development Medium (EDM; Walter et al., 2005), and for *P. halepensis*, the basal medium used was the DCR medium (Gupta and Durzan, 1985). Initiation and proliferation were carried out following the protocols described by Montalbán et al. (2012, 2013) for *P. radiata* and *P. halepensis*, respectively. Five established cell lines (ECLs) were used for each experiment and species.

### Amino Acid Supplementation Experiment

The maturation of *P. halepensis* and *P. radiata* EMs was carried out following the method described in Montalbán et al. (2010, 2013), respectively. Briefly, the EMs were suspended in a liquid basal medium without plant growth regulators and then filtered on a filter paper in a Büchner funnel. Aliquots containing 0.08 g of EMs on the filter papers were placed on each maturation media. The basal media (DCR for *P. halepensis* and EDM for *P. radiata*) were supplemented with 175 mM of sucrose, 9 gL^–1^ of Gelrite^®^, and 75 μM of abscisic acid (ABA) for *P. halepensis* or 60 μM of ABA for *P. radiata*; these maturation media were supplemented after autoclaving with different amino acid mixtures as described in Table 1: MIX I (Control) (Walter et al., 2005), MIX II – two times the concentration in the MIX I of the L-glutamine (Gln), L-asparagine (Asn), L-arginine (Arg), and L-proline (Pro); and MIX III – 4-fold the concentration in the MIX I of the Gln (Table 1).

**TABLE 1:** Different combinations of amino acids used in the maturation media (EDM and DCR) for *Pinus radiata* D. Don and *Pinus halepensis* Mill.

Amino acids	Treatments (mgL^–1^)		
	MIX I (Control)	MIX II	MIX III
L-glutamine	550	1100	2200
L-asparagine	525	1050	525
L-arginine	175	350	175
L-proline	17.5	35	17.5
L-citrulline	19.75	19.75	19.75
L-ornithine	19	19	19
L-lysine	13.75	13.75	13.75
L-alanine	10	10	10

Both, *P. radiata* and *P. halepensis* cultures were kept at 23°C and in darkness for 16 weeks on maturation media.

The germination of cotyledonary ses and the acclimatization of plantlets were performed according to Montalbán and Moncaleán (2019). Briefly, the ses were germinated for 8 weeks on half macronutrients LP [Quoirin and Lepoivre (1977), modified by Aitken-Christie et al. (1988)], supplemented with 2 gL^–1^ of activated charcoal and 9 gL^–1^ of Difco Agar granulated (Becton and Dickinson). First, petri dishes (90 mm × 15 mm) were used as containers, with the root caps of the ses pointing downward at an angle of approximately 60°. After this, the germinated plantlets were subcultured in the same medium for another month, but, in EcoBox^®^ (Eco2Box/green filter: a polypropylene vessel with a “breathing” hermetic cover, 125 mm × 65 mm × 80 mm, Duchefa). The cultures were kept at 23°C under 16-h photoperiod at 120 μmolm^–2^ s^–1^ provided by cool white fluorescent tubes (TFL 58 W/33; Philips, France) (Montalbán and Moncaleán, 2019).

After the germination, the plantlets were acclimatized in 43-cm^3^ pots, containing blond peat moss (Pindstrup, Ryomgård, Denmark): vermiculite (8:2, v/v) in the greenhouse under controlled conditions.

### Carbohydrate Supplementation Experiment

*Pinus radiata* and *P. halepensis* EMs obtained from the same materials and methods as in the amino acid supplementation experiment were used. The basal media described in amino acid supplementation experiment were supplemented with the MIX I (Table 1), and two different carbohydrate sources at two concentrations (four treatments) were assayed: 175 mM of sucrose (175 suc – control); 175 mM of maltose (175 mal); 350 mM of sucrose (350 suc), or 350 mM of maltose (350 mal). Cultures were kept at 23°C and in darkness for 16 weeks. Germination and acclimatization followed the same methodology described in the material and methods of the amino acid supplementation experiment.

### Osmolality of the Maturation Media

The osmolality (mosmkg^–1^) of all maturation media was measured at the onset of the experiment using a Micro-Osmometer Automatic (Löser Messtechnik, Berlin, Germany) according to the manufacturer’s instructions. Aliquots containing 100 μl of each maturation medium without Gelrite^®^ were measured, and the measurements were replicated three times (Montalbán et al., 2010).

### Free Polyamine Content Determination

Germinated ses obtained after 2 weeks in germination media were analyzed for free PA content for *P. radiata* (amino acid supplementation experiment) and *P. halepensis* (amino acid supplementation experiment and carbohydrate supplementation experiment). Germinated ses of *P. radiata* were not analyzed for the carbohydrate supplementation experiment, because we only obtained viable germinated ses for the control treatment. PA quantification was carried out according to Silveira et al. (2004) with some modifications. Samples (200 mg of fresh weight) were briefly grounded in 1.4 ml of 5% perchloric acid (v/v) in a Precellys^®^ shaker. After 1 h, the extraction solution was centrifuged for 20 min (15,000 *g*, 4°C), and supernatant was collected. The pellet was once again suspended in 0.2 ml of perchloric acid and centrifuged for 20 min (15,000 *g*, 4°C). Both extraction solutions were merged, homogenized, and frozen at −20°C for future analysis. Derivatization was performed according to Silveira et al. (2004), where 40 μl of each sample was mixed with 20 μl of diaminoheptane 0.05 mM, 50 μl of saturated sodium carbonate solution, and 100 μl of dansyl chloride in acetone 1.8 mM. After 50 min of incubation in the dark at 70°C, 25 μl of proline was added to the solution, followed by another 30-min incubation at room temperature. After that, 200 μl of toluene was added, the solution was vigorously shaken, and 175 μl of the superior organic phase with PAs was taken to a SpeedVac freeze dryer for 40 min at 40°C. Finally, pellets were suspended in 175 μl of acetonitrile and were ready for high-performance liquid chromatography (HPLC) quantification. Mobile phases were composed of (A) 10% (v/v) acetonitrile/ultrapure water pH 3.5 adjusted with HCl and (B) 100% of acetonitrile. The gradient started at 65% of B and lasted for 11 min; it was raised to 100% B for up to 25 min, maintained in 100% B until 35.5 min and then returned to 65% B until the end at 44 min. The flow rate was constantly 1 ml min^–1^, and oven temperature was maintained at 40°C. The stationary phase was a 5-μm Shim-pack CLC-ODS (M) 100 Å column with 250 mm × 4.6 mm equipped with a pre-column, both from Shimadzu^®^. The analyses were carried out in a Shimadzu Prominence HPLC equipped with a fluorescent detector configured to excitation of 340 nm and emission of 510-nm wavelengths. To determine PA concentration, peak areas of 20 μl samples were compared to triplicates of peak areas of correspondent standards of Put, Spd, Cad, and Spm, bought from Sigma-Merck^®^. The 1,7-diaminoheptane (DAH) (Sigma-Merck^®^) was used as an internal standard. Total free PAs were obtained by the sum of the individual PAs. Moreover, based on the fact that Put is a precursor to Spd and Spm (Mustafavi et al., 2018), the Put/(Spd + Spm) ratio was calculated. The final concentration of PAs was expressed in mmolμg^–1^.

### Data Collection and Statistical Analysis

For both species *P. radiata* and *P. halepensis*, the number of normal (NNE) (white to yellowish, non-germinating, with a distinct hypocotyl region, and at least three cotyledons) and abnormal mature ses (NAE) (germinating precociously, with fewer than three cotyledons or bearing abnormally shaped cotyledons) (Montalbán et al., 2010) per gram of embryogenic tissue was recorded. Also, the length (mm) (LE) and width (mm) (WE) of 240 (Amino acid supplementation experiment on each species) and 320 (Carbohydrate supplementation experiment on each species) NNE, and the LE/WE ratio of NNE were calculated. Embryos were obtained in three of the five maturated lines in both experiments and species. From the total of ses obtained, one half of them was germinated for the morphological analysis and the other half was germinated for the determination of free PA content. After 2 months in a germination medium, the germination rate for the NNE was calculated. Before *ex vitro* planting, the length of plantlets (mm), the length of aerial part (mm), the width of needles (mm), the stem diameter (mm), and the root length (mm) were measured with a digital caliber, and the number of secondary roots was counted in 10 plantlets per treatment. The percentage of acclimatization was calculated after 2 months in *ex vitro* conditions.

For Amino acid supplementation experiment, three different amino acid mixtures were tested in five ECLs for each species (R2, R9, R54, R82, and R137 for *P. radiata* and H48, H51, H149, H153, and H204 for *P. halepensis*) in a factorial with four repetitions (plates) per maturation condition and embryogenic cell line. In carbohydrate supplementation experiment, four different carbohydrates sources and concentrations were tested in five ECLs for *P. radiata* (R2, R9, R54, R82, and R137) and for *P. halepensis* (H48, H51, H149, H153, and H204) in a factorial with four repetitions.

For the effect of the ECLs on each of the variables of this study, an analysis of deviance was performed, followed by a Tukey *post hoc* test (α = 0.05), adjusted for multiple comparisons. To obtain robust conclusions, ECL factors were introduced into all the models as a block variable to reduce variability and analyze the effect of the culture medium more accurately.

In Amino acid supplementation experiment, the width of needles and stem diameter, for *P. radiata*, and *P. halepensis*, respectively, were square root transformed, and an ANOVA was performed. Multiple comparisons were made using the Tukey *post hoc* tests (α = 0.05). The same occurred in the carbohydrate supplementation experiment, for *P. halepensis*, in length of plantlets, width of needles, and the stem diameter.

In the amino acid supplementation experiment, for *P. radiata*, the NNE, length of plantlets, stem diameter, root part length, and number of secondary roots did not fulfill homoscedasticity and normality assumptions for ANOVA, and therefore, a Kruskal–Wallis was performed. The same occurred for the characteristics: *P. halepensis*—NNE, NAE, length of normal embryo, width of normal embryo, width of needles, and number of secondary roots in the amino acid supplementation experiment; *P. radiata*—length of normal embryo and the LE/WE ratio of ses in the carbohydrate supplementation experiment; and *P. halepensis*—NNE, NAE, length of normal embryo, width of normal embryo, the LE/WE ratio of ses, root part length, and number of secondary roots in the carbohydrate supplementation experiment.

For free PA content determination, the data obtained were *log* transformed, and an ANOVA was performed, followed by Tukey *post hoc* tests (α = 0.05), adjusted for multiple comparisons.

The data were analyzed using R software^®^, version 3.6.1. (R Core Team, 2017).

## Results

### Osmolality of the Maturation Medium

Statistically significant differences in the osmolality were observed between all the culture media with different compositions of amino acids and carbohydrates (Table 2). The osmolalities of the maturation media (EDM and DCR) were significantly higher when they contained 350 mM of sucrose compared with other carbohydrate and amino acid treatments (Table 3). Both of the maturation media supplemented with 175 mM of maltose showed the lowest values of osmolality (Table 3). Statistically significant differences were observed when comparing the osmolality of MIX I (control) with the media supplemented with MIX II and MIX III (Table 3).

**TABLE 2:** Analysis of deviance for the osmolality (mosmkg**^–^**^1^ water) of the different maturation media (MM) for *Pinus radiata* D. Don (EDM medium) and *Pinus halepensis* Mill (DCR medium).

Treatment	df	EDM medium		DCR medium	
		*F*-value	*P*-value	*F*-value	*P*-value
MM	5	3751	≤0.05*	3388	≤0.05*

*Significant differences at *p* ≤ 0.05; df, degrees of freedom. The values in the table correspond to the Kruskal–Wallis test.

**TABLE 3:** Osmolality (mosmkg**^–^**^1^ water) of the maturation media (M ± *SE*) for *Pinus radiata* D. Don (EDM medium) and *Pinus halepensis* Mill. (DCR medium).

Treatment	EDM medium (± *SE*)	DCR medium (± *SE*)
Control (175 mM of sucrose + MIX I)	277 ± 0.00^d^	260 ± 0.67^d^
175 mM of maltose	240 ± 0.58^e^	226 ± 0.88^e^
350 mM of sucrose	484 ± 3.18^a^	499 ± 1.00^a^
350 mM of maltose	428 ± 2.08^b^	411 ± 2.65^b^
MIX II	294 ± 1.00^c^	278 ± 2.96^c^
MIX III	293 ± 0.88^c^	272 ± 0.33^c^

*Different letters within a column show significant differences in the means observed by a Kruskal–Wallis test. MIX I (Control) – 550 mgL^–1^ of L-glutamine (Gln), 525 mgL^–1^ of L-asparagine (Asn), 175 mgL^–1^ of L-arginine (Arg), 17.5 mgL^–1^ of L-proline (Pro), 19.75 mgL^–1^ of L-citrulline, 19 mgL^–1^ of L-ornithine, 13.75 mgL^–1^ of L-lysine, and 10 mgL^–1^ of L-alanine; MIX II – two times the concentration in the MIX I of the Gln, Asn, Arg, and Pro; and MIX III – 4-fold the concentration in the MIX I of the Gln. *SE*, standard error.*

### Amino Acid Supplementation Experiment

Regarding the amino acid mixture used in the maturation media, statistically significant differences were found for all the parameters evaluated in *P. radiata* (NAE, LE, WE, germination of ses, and acclimatization of plantlets) except for the NNE and LE/WE ratio (Table 4).

**TABLE 4:** Analysis of deviance for the effect of the different mixes of amino acids (MA) in the maturation stage in: the number of normal and abnormal somatic embryos per 0.08 g of embryonal masses, the morphological characteristics of normal embryos, the germination of somatic embryos, the morphological characteristics, and the acclimatization of plantlets of *Pinus radiata* D. Don and *Pinus halepensis* Mill., respectively.

Characteristics	Source	df	*Pinus radiata*		*Pinus halepensis*	
			*F*-value	*P*-value	*F*-value	*P*-value
Number of normal embryos	MA	2	3.85	>0.05^ns^	0.37^1^	>0.05^ns^
Number of abnormal embryos	MA	2	6.06	≤0.01**	0.98^1^	>0.05^ns^
Length of normal embryos	MA	2	6.29	≤0.01**	52.48^1^	≤0.05*
Width of normal embryos	MA	2	13.24	≤0.05*	51.21^1^	≤0.05*
Length/Width ratio (mm)	MA	2	18.40	>0.05^ns^	21.45	>0.05^ns^
Germination of somatic embryos	MA	2	16.61	≤0.001***	22.59	≤0.01**
Length of plantlets (mm)	MA	2	4.47	>0.05^ns^	0.58	>0.05^ns^
Length of aerial part (mm)	MA	2	0.59	>0.05^ns^	0.13	>0.05^ns^
Width of needles (mm)	MA	2	1.78	>0.05^ns^	5.48^1^	>0.05^ns^
Stem diameter (mm)	MA	2	10.12	≤0.05*	1.16	>0.05^ns^
Root part length (mm)	MA	2	2.73	>0.05^ns^	1.44	>0.05^ns^
Number of secondary roots	MA	2	7.11	≤0.05*	0.18^1^	>0.05^ns^
Acclimatization of plantlets	MA	2	56.85	≤0.001***	19.33	>0.05^ns^

*;

**;

*** Significant differences at *p* ≤ 0.05, *p* ≤ 0.01, or *p* ≤ 0.001, respectively;

*^*ns*,^ non-significant at *p* ≤ 0.05; MA, mixes of amino acids; df, degrees of freedom. ^1^The values in the table correspond to the Kruskal–Wallis test.*

In the case of *P. halepensis*, the different mixtures of amino acids produced statistically significant differences for the LE, WE, and germination rate of NNE (Table 4). However, the mixture of different amino acids did not show statistically significant differences for the NNE, NAE, the LE/WE ratio, and acclimatization of plantlets (Table 4).

**FIGURE 1:**
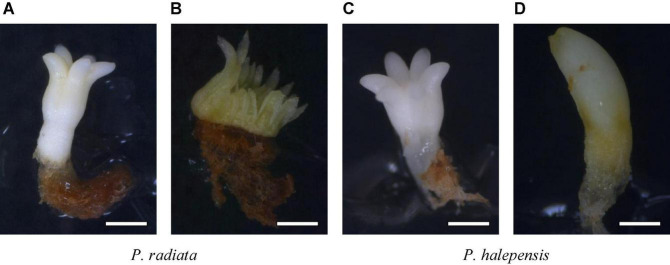
Somatic embryos showing different morphologies: *Pinus radiata* D. Don normal somatic **(A)**, bar = 1.44 mm; *P. radiata* abnormal somatic embryo **(B)**, bar = 1.85 mm; *Pinus halepensis* Mill. normal somatic embryos **(C)**, bar = 0.84 mm; *P. halepensis* abnormal somatic embryos **(D)**, bar = 0.60 mm.

**FIGURE 2:**
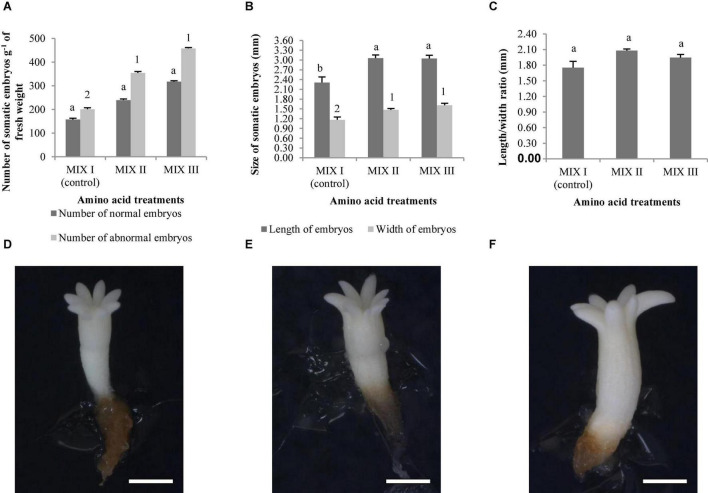
*Pinus radiata* D. Don somatic embryos of maturation media supplemented with different mixes of amino acids. Number of normal and abnormal somatic embryos **(A)**; the length and width of normal embryos **(B)**; and the length/width ratio of normal embryos **(C)**. Bars indicate standard errors. Significant differences at *p* ≤ 0.05 are indicated by different letters or numbers. *Pinus radiata* normal somatic obtained from maturation media supplemented with different mixes of amino acids. MIX I **(D),** bar = 1.85 mm; MIX II **(E)**, bar = 1.85 mm; and MIX III **(F)**, bar = 1.85 mm. MIX I (Control) – 550 mgL^–1^ of L-glutamine (Gln), 525 mgL^–1^ of L-asparagine (Asn), 175 mgL^–1^ of L-arginine (Arg), 17.5 mgL^–1^ of L-proline (Pro), 19.75 mgL^–1^ of L-citrulline, 19 mgL^–1^ of L-ornithine, 13.75 mgL^–1^ of L-lysine, and 10 mgL^–1^ of L-alanine; MIX II – two times the concentration in the MIX I of the Gln, Asn, Arg, and Pro; and MIX III – 4-fold the concentration in the MIX I of the Gln.

Figures 1A,B show normal and aberrant morphologies of ses of *P. radiata*, respectively. For *P. radiata*, although non-significant differences were found among the different combinations of amino acids when compared with the production of NNE (Table 4), an increase of the NNE in MIX II and MIX III (240 and 317 sesg^–1^ fresh weight, respectively), compared with the control (MIX I – 157 sesg^–1^ fresh weight), was observed (Figure 2A). The same tendency was found for the NAE, with a statistically significant increase for NAE in media supplemented with MIX II and MIX III when compared to MIX I (Figure 2A). Moreover, we observed that treatments MIX II and MIX III induced an increase in LE and WE when compared to control treatment (MIXI) (Figures 2B,D,E,F). With respect to the LE/WE ratio, non-significant differences were found between the different combinations of amino acids (Figure 2C).

**FIGURE 3:**
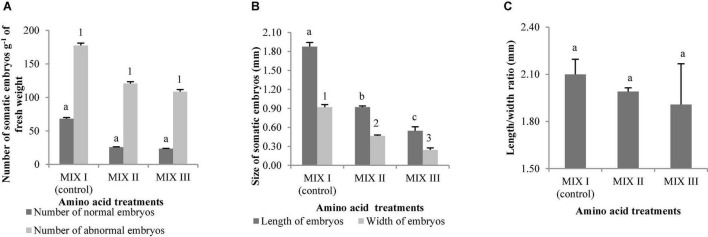
*Pinus halepensis* Mill. somatic embryos obtained per gram of embryonal masses matured in different mixes of amino acids. Number of normal and abnormal somatic embryos **(A)**; the length and width of normal embryos **(B)**; the length/width ratio of normal embryos **(C)**. Bars indicate standard errors. Significant differences at *p* ≤ 0.05 are indicated by different letters or numbers. MIX I (Control) – 550 mgL^–1^ of L-glutamine (Gln), 525 mgL^–1^ of L-asparagine (Asn), 175 mgL^–1^ of L-arginine (Arg), 17.5 mgL^–1^ of L-proline (Pro), 19.75 mgL^–1^ of L-citrulline, 19 mgL^–1^ of L-ornithine, 13.75 mgL^–1^ of L-lysine, and 10 mgL^–1^ of L-alanine; MIX II – two times the concentration in the MIX I of the Gln, Asn, Arg, and Pro; and MIX III – 4-fold the concentration in the MIX I of the Gln.

Figures 1C,D show normal and aberrant morphologies of ses of *P. halepensis*, respectively. For *P. halepensis*, although non-significant differences were found among the different mixtures of amino acids for NNE and NAE (Table 4), the highest NNE and NAE were recorded in control conditions (68 sesg^–1^ fresh weight for NNE and 178 sesg^–1^ fresh weight for NAE); MIX II showed intermediate values, and MIX III presented the lowest values (23 sesg^–1^ fresh weight for NNE and 109 sesg^–1^ fresh weight for NAE) (Figure 3A).

*Pinus halepensis* ses developed in control conditions (MIX I) were significantly wider and longer than those obtained in MIX II and MIX III. Significantly lower LE and WE values were obtained when the maturation medium was supplemented with a 4-fold concentration of Gln (MIX III) (Figure 3B), although the LE/WE ratio did not show significant differences (Figure 3C).

**FIGURE 4:**
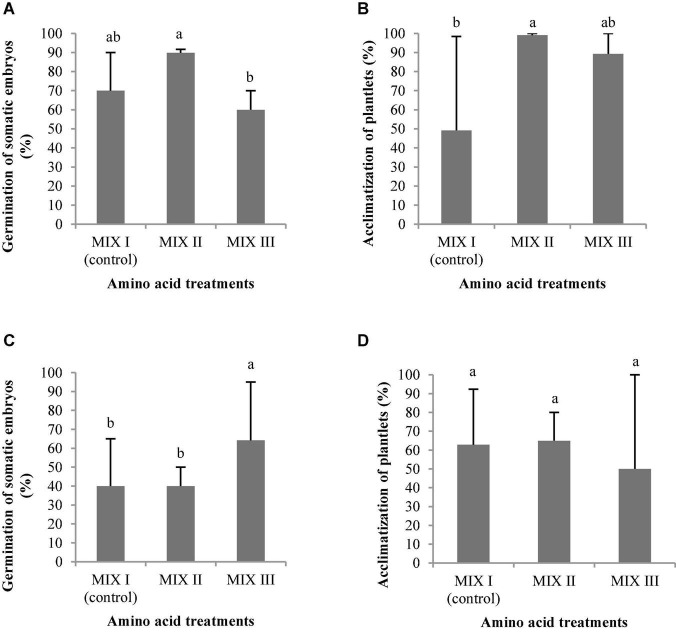
*Pinus radiata* D. Don germination (%) of somatic embryos maturated in a medium supplemented with different mixes of amino acids **(A)**. Acclimatization (%) of plantlets of *P. radiata* obtained from somatic embryos maturated in a medium supplemented with different mixes of amino acids **(B)**. Germination (%) of somatic embryos of *Pinus halepensis* Mill. maturated in a medium supplemented with different mixes of amino acids **(C)**. Acclimatization (%) of plantlets of *P. halepensis* obtained from somatic embryos maturated in a medium supplemented with different mixes of amino acids **(D)**. Bars indicate standard errors. Significant differences at *p* ≤ 0.05 are indicated by different letters. MIX I (Control) – 550 mgL^–1^ of L-glutamine (Gln), 525 mgL^–1^ of L-asparagine (Asn), 175 mgL^–1^ of L-arginine (Arg), 17.5 mgL^–1^ of L-proline (Pro), 19.75 mgL^–1^ of L-citrulline, 19 mgL^–1^ of L-ornithine, 13.75 mgL^–1^ of L-lysine, and 10 mgL^–1^ of L-alanine; MIX II – two times the concentration in the MIX I of the Gln, Asn, Arg, and Pro; and MIX III – 4-fold the concentration in the MIX I of the Gln.

**FIGURE 5:**
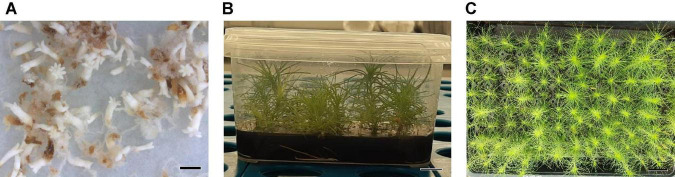
Maturation, germination, and acclimatization of somatic embryos from *Pinus radiata* D. Don embryogenic cell lines. Somatic embryos after 4 months in a maturation medium **(A)**, bar = 3 mm; somatic plantlets after 3 months in a germination medium **(B)**, bar = 1.4 cm; plantlets derived from normal cotyledonary somatic embryos growing in the greenhouse **(C)**, bar = 2.35 cm. The maturation medium was supplemented with MIX I (Control) – 550 mgL^–1^ of L-glutamine (Gln), 525 mgL^–1^ of L-asparagine (Asn), 175 mgL^–1^ of L-arginine (Arg), 17.5 mgL^–1^ of L-proline (Pro), 19.75 mgL^–1^ of L-citrulline, 19 mgL^–1^ of L-ornithine, 13.75 mgL^–1^ of L-lysine, and 10 mgL^–1^ of L-alanine; MIX II – two times the concentration in the MIX I of the Gln, Asn, Arg, and Pro; and MIX III – 4-fold the concentration in the MIX I of the Gln.

As observed in Figure 4A, the best germination percentage of *P. radiata* was obtained in those embryos coming from ECLs maturated on a medium supplemented with double the concentration of Gln, Asn, Arg, and Pro (MIX II), but not in relation to control treatment (MIX I). However, the treatment with 4-fold of Gln (MIX III) promoted a significant decrease of the ses germination with respect to MIX II (Figure 4A). Plantlets of *P. radiata* survived after acclimatization with significantly higher percentage of acclimatization when coming from ses maturated on a culture medium supplemented with MIX II (99%) and MIX III (89.29%) than compared with control (MIX I) (49.21%) (Figures 4B, 5).

*Pinus halepensis* germination significantly increased in those embryos coming from ECLs maturated in the presence of 4-fold the concentration of Gln (MIX III) (64%) when compared to results obtained with other amino acid combinations (Figure 4C). Significant differences were not observed for the survival of plantlets in the greenhouse (62.82, 65, and 50% for MIX I, MIX II, and MIX III, respectively) (Figure 4D).

**FIGURE 6:**
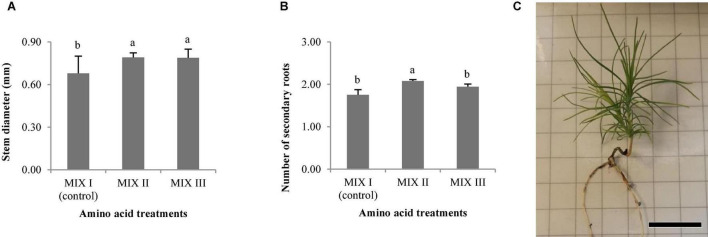
Morphological characteristics of *Pinus radiata* D. Don plantlets, stem diameter (mm) **(A)**, and number of secondary roots **(B)**. Bars indicate standard errors. Significant differences at *p* ≤ 0.05 are indicated by different letters or numbers. Plantlet obtained from somatic embryos maturated in EDM medium with MIX II **(C)**, bar = 2 cm. MIX I (Control) – 550 mgL^–1^ of L-glutamine (Gln), 525 mgL^–1^ of L-asparagine (Asn), 175 mgL^–1^ of L-arginine (Arg), 17.5 mgL^–1^ of L-proline (Pro), 19.75 mgL^–1^ of L-citrulline, 19 mgL^–1^ of L-ornithine, 13.75 mgL^–1^ of L-lysine and 10 mgL^–1^ of L-alanine; MIX II, twice the concentration in the MIX I of the Gln, Asn, Arg, and Pro; MIX III, fourfold the concentration in the MIX I of the Gln.

Statistically significant differences for the stem diameter and the number of secondary roots were observed in *P. radiata* (Table 4). The mean length of the plantlets ranged from 38.13 to 47.66 mm, and this value was divided into the aerial part and the root part (Table 5). In this sense, the width of the needle varied between 0.27 and 0.32 mm (Table 5). The MIX II and MIX III promoted a significant increase of the stem diameter when compared to the control (Figure 6A). The same tendency was observed with the number of secondary roots where the MIX II promoted a significant increase in the number of secondary roots (Figures 6B,C).

**TABLE 5:** Morphological characteristics of *P. radiata* D. Don somatic plantlets developed from ECLs maturated in a culture medium supplemented with different amino acid compositions (M ± SE).

Morphological characteristics	MIX I – control	MIX II	MIX III
Length of plantlets (mm)	38.136.72	47.175.83	47.665.86
Length of aerial part (mm)	6.130.59	6.910.52	6.280.48
Width of needles (mm)	0.280.32	0.320.02	0.270.01
Root length (mm)	26.584.04	20.684.69	24.404.66

*MIX I (Control) – 550 mgL^–1^ of L-glutamine (Gln), 525 mgL^–1^ of L-asparagine (Asn), 175 mgL^–1^ of L-arginine (Arg), 17.5 mgL^–1^ of L-proline (Pro), 19.75 mgL^–1^ of L-citrulline, 19 mgL^–1^ of L-ornithine, 13.75 mgL^–1^ of L-lysine, and 10 mgL^–1^ of L-alanine; MIX II – two times the concentration in the MIX I of the Gln, Asn, Arg, and Pro; and MIX III – 4-fold the concentration in the MIX I of the Gln. *SE*, standard error.*

When evaluating the morphological characteristics of the germinated ses developed from ECLs of *P. halepensis* maturated in the presence of different combinations of amino acids, no statistically significant differences were observed (Tables 4, 6).

**TABLE 6:** Morphological characteristics of *Pinus halepensis* Mill. somatic plantlets developed from ECLs maturated in a culture medium supplemented with different amino acid compositions (M ± SE).

Morphological characteristics	MIX I – control	MIX II	MIX III
Length of plantlets (mm)	77.9611.50	97.6315.37	92.9812.76
Length of aerial part (mm)	6.941.24	7.581.09	6.821.05
Width of needles (mm)	0.310.03	0.270.03	0.230.01
Stem diameter (mm)	0.680.05	0.790.06	0.790.07
Root length (mm)	38.0910.23	64.6413.27	57.1910.47
Number of secondary roots	20.36	20.31	20.29

*MIX I (Control) – 550 mgL^–1^ of L-glutamine (Gln), 525 mgL^–1^ of L-asparagine (Asn), 175 mgL^–1^ of L-arginine (Arg), 17.5 mgL^–1^ of L-proline (Pro), 19.75 mgL^–1^ of L-citrulline, 19 mgL^–1^ of L-ornithine, 13.75 mgL^–1^ of L-lysine, and 10 mgL^–1^ of L-alanine; MIX II – two times the concentration in the MIX I of the Gln, Asn, Arg, and Pro; and MIX III – 4-fold the concentration in the MIX I of the Gln. *SE*, standard error.*

### Carbohydrate Supplementation Experiment

The carbohydrate sources significantly affected the NNE, the NAE, and the LE (Table 7). For NAE, WE, and the LE/WE ratio, a significant interaction between the carbohydrate sources and concentrations was observed (Table 7). Finally, the carbohydrate source and the carbohydrate concentration had a significant effect on the germination rates, but there was no interaction between the previously mentioned factors (Table 7).

**TABLE 7:** Analysis of deviance for the effect of the different carbohydrate sources (CS) and concentrations (CC) in the maturation stage in the number of normal and abnormal somatic embryos per 0.08 g of embryonal masses; the morphological characteristics of normal embryos*;* the germination of somatic embryos of *Pinus radiata* D. Don and *Pinus halepensis* Mill., respectively*;* and the effect of the different CS in the germination of somatic embryos, the morphological characteristics, and the acclimatization of plantlets *P. halepensis*.

Characteristics	Source	df	*Pinus radiata*		*Pinus halepensis*	
			*F*-value	*P*-value	*F*-value	*P*-value
Number of normal embryos	CS	1	5.15	≤0.05*	2.80^1^	>0.05^ns^
	CC	1	0.67	>0.05^ns^	0.90	>0.05^ns^
	SC × CC	3	1.93	>0.05^ns^	2.80	>0.05^ns^
Number of abnormal embryos	CS	1	11.30	≤0.01**	16.89^1^	≤0.001***
	CC	1	1.45	>0.05^ns^	0.27	>0.05^ns^
	SC × CC	3	7.48	≤0.01**	16.90	≤0.001***
Length of normal embryos	CS	1	4.22^1^	≤0.05*	23.44^1^	≤0.05*
	CC	1	0.78	>0.05^ns^	31.57	≤0.05*
	SC × CC	3	4.25	>0.05^ns^	28.13	≤0.05*
Width of normal embryos	CS	1	0.44	>0.05^ns^	32.10^1^	≤0.001***
	CC	1	0.65	>0.05^ns^	20.19	≤0.001***
	SC × CC	3	20.17	≤0.01**	35.21	≤0.001***
Length/Width ratio (mm)	CS	1	5.86^1^	≤0.05*	12.88^1^	≤0.05*
	CC	1	1.08	>0.05^ns^	3.16	>0.05^ns^
	SC × CC	3	10.14	≤0.05*	17.21	≤0.05*
Germination of somatic embryos	CS	1	36.69	≤0.001***	82.11	≤0.001***
	CC	1	0.51	≤0.001***	–	–
	SC × CC	3	0.51	>0.05^ns^	–	–
Length of plantlets (mm)	CS	1	–	–	1.21	>0.05^ns^
Length of aerial part (mm)	CS	1	–	–	0.89	≤0.05*
Width of needles (mm)	CS	1	–	–	0.47	>0.05^ns^
Stem diameter (mm)	CS	1	–	–	1.07	>0.05^ns^
Root part length (mm)	CS	1	–	–	2.56^1^	>0.05^ns^
Number of secondary roots	CS	1	–	–	4.76^1^	≤0.05*
Acclimatization of plantlets	CS	1	–	–	20.15	≤0.001***

*;

**;

*** Significant differences at *p* ≤ 0.05, *p* ≤ 0.01, or *p* ≤ 0.001, respectively;

*^*ns*,^ non-significant at *p* ≤ 0.05; df, degrees of freedom. ^1^The values in the table correspond to the Kruskal–Wallis test.*

**FIGURE 7:**
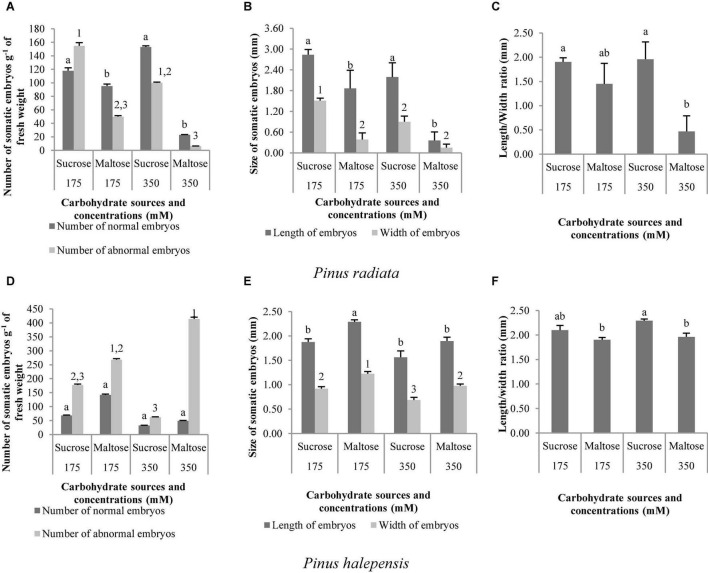
*Pinus radiata* D. Don and *Pinus halepensis* Mill. somatic embryos obtained per gram of embryonal masses matured in different carbohydrate sources and concentrations. Number of normal and abnormal somatic embryos **(A,D)**; the length and width of normal embryos **(B,E)**; and the length/width ratio of normal embryos **(C,F)**. Bars indicate standard errors. Significant differences at *p* ≤ 0.05 are indicated by different letters or numbers.

Two types of carbohydrates in both concentrations produced normal and aberrant embryos in *P. radiata*. However, the use of sucrose in the maturation medium, regardless of the concentration, resulted in a significantly higher NNE (Figure 7A). In this regard, we also observed the highest NAE in the medium of maturation with sucrose but only at a concentration of 175 suc (control) in relation to maltose (Figure 7A). In addition, the same tendency was observed for LE and WE (Figure 7B), with the development of longer and wider ses in the maturation medium with sucrose, regardless of the concentration. The ses maturated in media with sucrose presented a significantly higher LE/WE ratio than those obtained from a medium with 350 mal (Figure 7C).

In *P. halepensis*, the NNE was not affected by the different carbohydrate treatments tested (Table 7 and Figure 7D). However, NAE, LE, WE, and the LE/WE ratio were significantly affected by the interaction between the carbohydrate source and the concentration (Table 7). The medium with the highest maltose concentration produced a significantly higher number of NAE than the sucrose media (Figure 7D).

**FIGURE 8:**
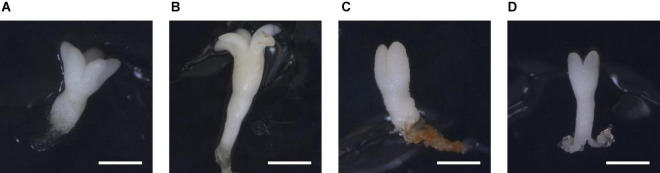
Somatic embryos of *Pinus halepensis* Mill. maturated in the DCR medium supplemented with: 175 mM of sucrose **(A)**, bar = 1 mm; 175 mM of maltose **(B)**, bar = 1.57 mm; 350 mM of sucrose **(C)**, bar = 0.57 mm; and 350 mM of maltose **(D)**, bar = 1 mm.

The LE and the WE of *P. halepensis* obtained in a maturation medium supplemented with 175 mal were significantly higher than the LE obtained after maturation with other carbohydrate sources or concentrations (Figure 7E). As noted in Figure 7F, a significantly higher LE/WE ratio was observed in ses maturated with the highest concentration of 350 suc when compared to results obtained in ses from media supplemented with maltose. Thus, higher concentrations of carbohydrates caused the formation of less developed embryos (Figure 8).

**FIGURE 9:**
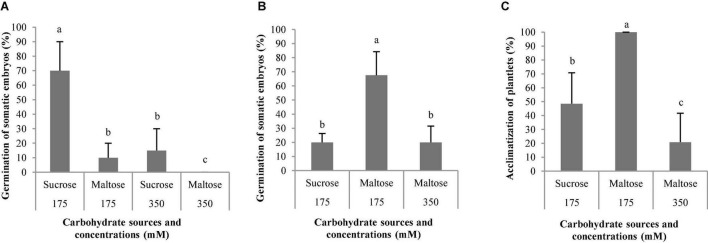
Germination (%) of somatic embryos of *Pinus radiata* D. Don maturated in a medium supplemented with different carbohydrate sources and concentrations **(A)**. Germination (%) of somatic embryos of *Pinus halepensis* Mill. maturated in a medium supplemented with different carbohydrate sources and concentrations **(B)**. Acclimatization (%) of plantlets of *P. halepensis* obtained from somatic embryos maturated in a medium supplemented with different carbohydrate sources and concentrations **(C)**. Bars indicate standard errors. Significant differences at *p* ≤ 0.05 are indicated by different letters.

The use of 175 suc promoted a germination percentage of 70%, while the other treatments promoted a percentage lower than 15% for ses of *P. radiata* (Figure 9A). However, the ses maturated at a maturation medium with a high concentration of maltose did not promote the germination of ses (Figure 9A). Viable plantlets were only obtained in those embryos germinated in culture media with 175 suc where the acclimatization percentage was 49.21%.

**FIGURE 10:**
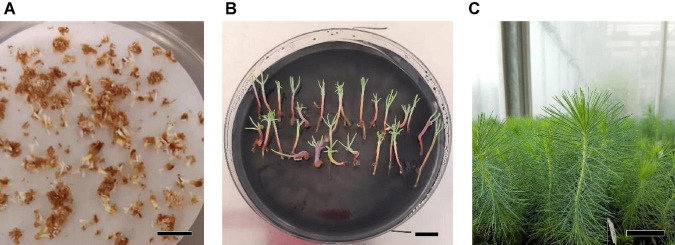
Maturation, germination, and acclimatization of somatic embryos from *Pinus halepensis* Mill. embryogenic cell lines. Somatic embryos after 4 months in a maturation medium **(A)**, bar = 1 cm; somatic plantlets after 3 months cultivated in a germination medium **(B)**, bar = 1 cm; plantlets derived from normal cotyledonary somatic embryos growing in the greenhouse **(C)**, bar = 3 cm. The maturation medium was supplemented with MIX I (Control) – 550 mgL^–1^ of L-glutamine (Gln), 525 mgL^–1^ of L-asparagine (Asn), 175 mgL^–1^ of L-arginine (Arg), 17.5 mgL^–1^ of L-proline (Pro), 19.75 mgL^–1^ of L-citrulline, 19 mgL^–1^ of L-ornithine, 13.75 mgL^–1^ of L-lysine, and 10 mgL^–1^ of L-alanine; MIX II – two times the concentration in the MIX I of the Gln, Asn, Arg, and Pro; and MIX III – 4-fold the concentration in the MIX I of the Gln.

The ses of *P. halepensis* maturated with 175 mal showed a germination percentage significantly (Table 7) higher than the rest of the treatments assayed (Figure 9B). However, the ses maturated with 350 suc did not germinate. The plantlets of *P. halepensis* from the maturation treatment with 175 mM of maltose survived after acclimatization in a significantly higher percentage (100%) than those that came from a treatment with 175 suc (control) (Table 7 and Figures 9C, 10). In contrast, the worst results were obtained in the treatment with a higher concentration of maltose (Figure 9C).

**FIGURE 11:**
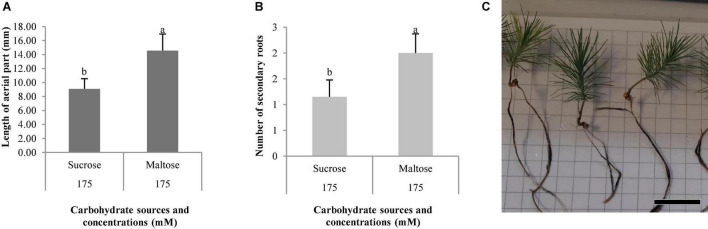
Morphological characteristics of *Pinus halepensis* Mill. plantlets, length of aerial part (mm) **(A)**, and number of secondary roots **(B)**. Bars indicate standard errors. Significant differences at *p* ≤ 0.05 are indicated by different letters or numbers. Plantlets of *Pinus halepensis* Mill. obtained from somatic embryos maturated in the EDM medium supplemented with 175 mM of maltose **(C)**, bar = 3 cm.

The length of aerial part and number of secondary roots were significantly affected by the different carbohydrate sources, and no differences were observed for the other morphological characteristics studied in *P. halepensis* (Table 7). It was observed that 175 mal promoted an increase in the aerial part and number of secondary roots in comparison with 175 suc (Figures 11A,B). Although significantly statistical differences were not found for the other morphological characteristics, an increase in the length of plantlets was observed in those obtained from the ses developed in a maturation medium supplemented with 175 mal (Table 8 and Figure 11C). The same results were observed for a stem diameter and the root length; but the largest width of the needle was observed in 175 suc (Table 8).

**TABLE 8:** Morphological characteristics of *Pinus halepensis* Mill. somatic plantlets developed from ECLs maturated in a culture medium supplemented with different carbohydrate sources.

Morphological characteristics	175 mM of sucrose	175 mM of maltose
Length of plantlets (mm)	59.29 ± 10.57	74.19 ± 9.80
Width of needles (mm)	0.27 ± 0.03	0.25 ± 0.02
Stem diameter (mm)	0.66 ± 0.03	0.75 ± 0.06
Root length (mm)	28.24 ± 7.84	44.69 ± 8.25

*SE, standard error.*

### Free Polyamine Content Determination

**FIGURE 12:**
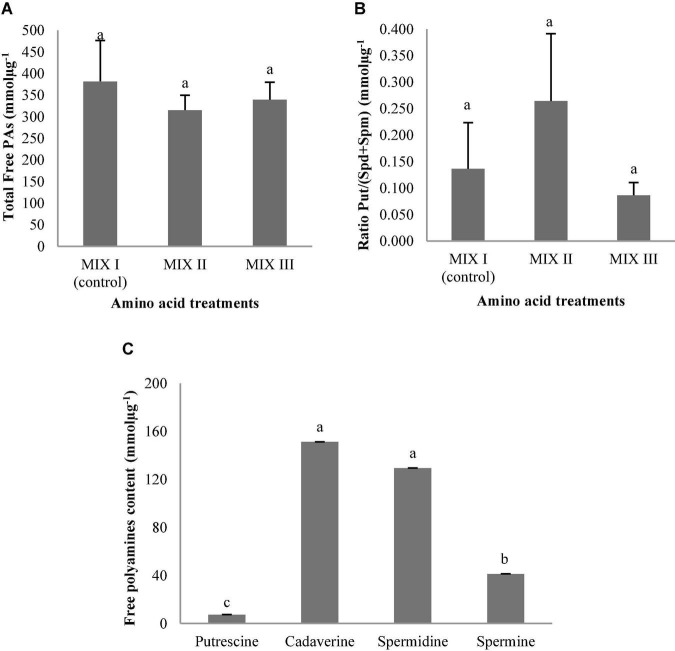
Polyamine (PA) levels (mmolμg^–1^) in germinated somatic embryos of *Pinus radiata* D. Don obtained from somatic embryos maturated in media supplemented with different mixes of amino acids. Total Free PAs **(A)**; the ratio Put/(Spd + Spm) **(B)**; and the individual free PAs **(C)**. Bars indicate standard errors. Significant differences at *p* ≤ 0.05 are indicated by different letters. MIX I (Control) – 550 mgL^–1^ of L-glutamine (Gln), 525 mgL^–1^ of L-asparagine (Asn), 175 mgL^–1^ of L-arginine (Arg), 17.5 mgL^–1^ of L-proline (Pro), 19.75 mgL^–1^ of L-citrulline, 19 mgL^–1^ of L-ornithine, 13.75 mgL^–1^ of L-lysine, and 10 mgL^–1^ of L-alanine; MIX II – two times the concentration in the MIX I of the Gln, Asn, Arg, and Pro; and MIX III – 4-fold the concentration in the MIX I of the Gln.

The addition of different combinations of amino acids in the maturation medium did not provoke statistically significant differences in the ses germination of *P. radiata*, as well as no statistically significant differences were observed for the interaction between amino acid mixtures and the amounts of PAs (Table 9). However, the only statistically significant differences were found when the content of individual Put, Cad, Spd, and Spm was analyzed (Table 9). Conversely, total free PAs and the ratio Put/(Spd + Spm) did not show significant differences (Table 9 and Figures 12A,B). A higher amount of total free PAs was observed, with means ranging from 300.09 to 368.80 mmolμg^–1^ (Figure 12A). Within the total Free PAs, Cad accounted for half the amount of the total quantity followed by Spd (Figure 12C). However, the ses germinated from *P. radiata* had three times less Spm when compared with the other PAs previously mentioned, while the amount of Put was the lowest free PAs detected (Figure 12C). Plantlets of *P. radiata* contained large amounts of Cad and Spd, intermediate amounts of Spm, and low amounts of Put (Figure 12C).

**TABLE 9:** Analysis of variance for the effect of the mixes of amino acids (MA) and the free individual polyamine levels (PAs); and for the effect of the mixes of MA on the total free Pas and the ratio Put/(Spd + Spm) in germinated somatic embryos of *Pinus radiata* D. Don and *Pinus halepensis* Mill.

PAs content	Source	df	*Pinus radiata*		*Pinus halepensis*	
			*F*-value	*P*-value	*F*-value	*P*-value
Free PAs (mmolμg^–1^)	MA	2	0.68	>0.05^ns^	1.04	>0.05^ns^
	PAs	3	190.99	≤0.001***	54.04	≤0.001***
	MA × PAs	6	0.44	>0.05^ns^	3.21	≤0.05*
Total free PAs (mmolμg^–1^)	MA	2	0.49	>0.05^ns^	0.76	>0.05^ns^
Ratio Put/(Spd + Spm) (mmolμg^–1^)	MA	2	0.28	>0.05^ns^	3.23	>0.05^ns^

*;

*** Significant differences at *p* ≤ 0.05 or *p* ≤ 0.001, respectively;

*^*ns*,^ non-significant at *p* ≤ 0.05; df, degrees of freedom; Put, putrescine; Spd, spermidine; Spm, spermine. Mixes of amino acids: MIX I (Control) – 550 mgL^–1^ of L-glutamine (Gln), 525 mgL^–1^ of L-asparagine (Asn), 175 mgL^–1^ of L-arginine (Arg), 17.5 mgL^–1^ of L-proline (Pro), 19.75 mgL^–1^ of L-citrulline, 19 mgL^–1^ of L-ornithine, 13.75 mgL^–1^ of L-lysine, and 10 mgL^–1^ of L-alanine; MIX II – two times the concentration in the MIX I of the Gln, Asn, Arg, and Pro; and MIX III – 4-fold the concentration in the MIX I of the Gln.*

**FIGURE 13:**
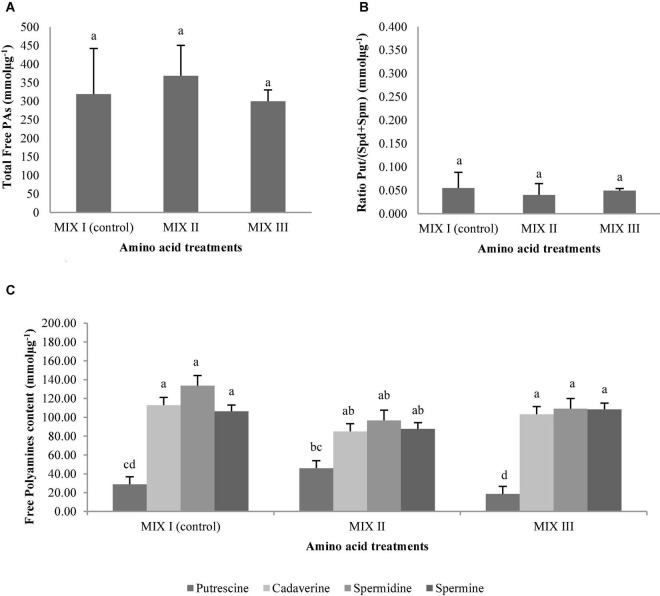
Polyamine (PAs) levels (mmolμg^–1^) in germinated somatic embryos of *Pinus halepensis* Mill. obtained from somatic embryos maturated in media supplemented with different mixes of amino acids. Total Free PAs **(A)**; the ratio Put/(Spd + Spm) **(B)**; and the individual free PAs **(C)**. Bars indicate standard errors. Significant differences at *p* ≤ 0.05 are indicated by different letters. MIX I (Control) – 550 mgL^–1^ of L-glutamine (Gln), 525 mgL^–1^ of L-asparagine (Asn), 175 mgL^–1^ of L-arginine (Arg), 17.5 mgL^–1^ of L-proline (Pro), 19.75 mgL^–1^ of L-citrulline, 19 mgL^–1^ of L-ornithine, 13.75 mgL^–1^ of L-lysine, and 10 mgL^–1^ of L-alanine; MIX II – two times the concentration in the MIX I of the Gln, Asn, Arg, and Pro; and MIX III – 4-fold the concentration in the MIX I of the Gln.

Statistically significant differences were observed in PAs and the interaction between the amino acid mixtures and free PAs in germinated ses of *P. halepensis* (Table 9). However, no statistically significant differences were observed for the total free PAs and the ratio Put/(Spd + Spm) (Table 9 and Figures 13A,B). The highest concentrations of total free PAs were observed in ses from MIX II treatment (Figure 13A). The amounts of Cad, Spd, and Spm did not change during the germination of ses obtained from maturation media supplemented with different combinations of amino acids (Figure 13C). The level of these three PAs exhibited a slight increase in MIX I compared to the other treatments (Figure 13C). In this case, the amount of Spd increased slightly, followed by Cad (Figure 13C). In contrast, the Put concentration exhibited a considerable significant decrease in MIX I and MIX III (Figure 13C). Exogenous application of amino acids of MIX II in the maturation medium promoted similar amounts of four Pas analyzed in the germinated ses (Figure 13C).

For *P. halepensis*, statistically significant differences were observed for the different sources of carbohydrates, PAs, the interaction between carbohydrate sources, and carbohydrate concentrations and the triple interaction carbohydrate sources, carbohydrate concentrations, and PAs (Table 10).

**TABLE 10:** Analysis of variance for the effect of the different carbohydrates sources (CS) and concentration (CC), and the free polyamine levels (PAs) (mmolμg^–1^); and for the effect of the different carbohydrate sources (CS) and concentration (CC) in the total free polyamine (PAs) (mmolμg^–1^) and the ratio Put/(Spd + Spm) in germinated somatic embryos of *Pinus halepensis* Mill.

PAs content	Source	df	*F*-value	*P*-value
Free PAs	CS	1	7.58	≤0.001***
	CC	1	0.88	>0.05^ns^
	PAs	3	38.17	≤0.001***
	SC × CC	1	4.22	≤0.05*
	CS × PAs	3	2.21	>0.05^ns^
	CC × PA	3	2.40	>0.05^ns^
	CS × CC × PAs	3	10.28	≤0.001***
Total free PAs	CS	1	1.88	>0.05^ns^
	CC	1	0.00	>0.05^ns^
	SC × CC	1	0.10	>0.05^ns^
Ratio Put/(Spd + Spm)	CS	1	0.00	>0.05^ns^
	CC	1	1.85	>0.05^ns^
	SC × CC	1	13.87	≤0.01**

*;

**;

*** Significant differences at *p* ≤ 0.05, *p* ≤ 0.01 or *p* ≤ 0.001, respectively;

*^*ns*^, non-significant at *p* ≤ 0.05; df, degrees of freedom; Put, putrescine; Spd, spermidine; Spm, spermine.*

**FIGURE 14:**
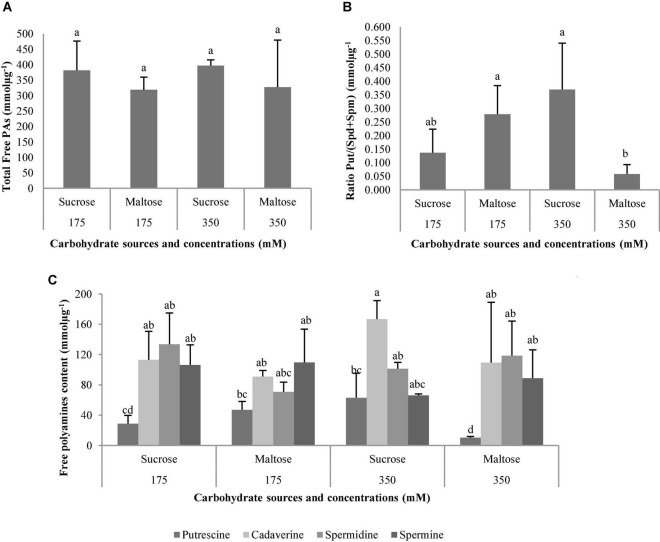
Polyamine (PAs) levels (mmolμg**^–^**^1^) in plantlets of *Pinus halepensis* Mill. obtained from germinated somatic embryos maturated in media supplemented with different carbohydrate sources and concentrations. Total Free Pas **(A)**; the ratio Put/(Spd + Spm) **(B)**; ant the Free Pas **(C)**. Bars indicate standard errors. Significant differences at *p* ≤ 0.05 are indicated by different letters.

The total free PAs were not affected by the carbohydrate sources, carbohydrate concentrations, or the interaction between these factors (Table 10 and Figure 14A). On the contrary, the ratio Put/(Spd + Spm) was affected by the interaction carbohydrate sources × carbohydrate concentration (Table 10). The *P. halepensis* germinated ses maturated in media with 350 suc or 175 mal showed significantly higher Put/(Spd + Spm) ratios than those obtained in a medium with the lowest maltose concentration (Figure 14B). Additionally, an intermediate value for Put/(Spd + Spm) ratios was observed in the germinated ses maturated in control conditions (175 suc) (Figure 14B). Germinated ses coming from maturation media with different carbohydrate sources and concentrations showed statistical higher values of all PAs, except Put (Figure 14C). In this case, levels of Put presented a marked decrease in germinated ses maturated in maturation media 350 mal when compared with the germinated ses maturated in maturation media 175 mal and 350 suc (Figure 14C). Cad, Spd, and Spm detected in germinated ses maturated in different carbohydrate sources and concentrations presented a similar behavior, but with an increase in the Cad amount in the germinated ses maturated in maturation media with 350 suc (Figure 14C).

## Discussion

Different amino acid or carbohydrate combinations significantly affected the osmolality of the maturation culture medium; it is common in the conifers SE process to restrict the water availability of the medium (increasing gellan gum concentration), modifying the osmolality in order to cause a shift in the developmental program of EMs and promote the formation of ses (Kvaalen and Johnsen, 2008; Garcia-Mendiguren et al., 2016). In our experiments, the best environment to achieve proper conversion of ses in plantlets was observed in maturation media with osmolalities between 277 (175 mM of sucrose) and 294 mosmkg^–1^ water (MIX II) for *P. radiata* and between 226 (175 mM of maltose) and 272 mosmkg^–1^ water (MIX III) for *P. halepensis*. However, and in agreement with Montalbán et al. (2010), who obtained good results at osmolalities around 270 mosmkg^–1^ water, the medium osmolality alone did not explain the results obtained because, in other media with similar osmolalities, such good results were not obtained in both species.

In our work, the highest osmolalities (above 400 mosmkg^–1^ water) did not increase the maturation success; in contrast, in *Pseudotsuga menziesii* (Mirb.) Franco raising medium osmolality to 450 mosmkg^–1^ water provoked an improvement in the vigor and morphology of ses developed (Gupta and Pullman, 1991). In this sense, the improvement in the number of ses in *P. radiata* and in *Cryptomeria japonica* D. Don was obtained in lower water availability in maturation media caused by 10 gL^–1^ of gellan gum and 17.5% of polyethylene glycol (PEG), respectively (Moncaleán et al., 2018; Maruyama et al., 2021). However, in *Pinus taeda* L., the maturation medium with 13% of PEG 8,000 and the osmolality ranging from 227 to 233 mmolkg^–1^ allowed an increase in the number of ses (Pullman et al., 2003).

When the size of the *P. radiata* somatic embryos was analyzed, we found that the maturation with MIX I (550 mgL^–1^ of Gln, 525 mgL^–1^ of Asn, 175 mgL^–1^ of L-Arg, 17.5 mgL^–1^ of Pro, 19.75 mgL^–1^ of L-citrulline, 19 mgL^–1^ of L-ornithine, 13.75 mgL^–1^ of L-lysine, and 10 mgL^–1^ of L-alanine) promoted the formation of smaller ses, showing lower acclimatization success months later (Figure 4B). In contrast, germinated ses maturated on MIX II (1,100 mgL^–1^ of Gln, 1,050 mgL^–1^ of Asn, 350 mgL^–1^ of L-Arg, 35 mgL^–1^ of Pro, 19.75 mgL^–1^ of L-citrulline, 19 mgL^–1^ of L-ornithine, 13.75 mgL^–1^ of L-lysine, and 10 mgL^–1^ of L-alanine) promoted larger embryos, with a larger stem diameter and an increase in the number of roots in the germinated ses, improving the acclimatization to *ex vitro* conditions of this species (Figure 4B). Similar to our results with *P. radiata*, Afele and Saxena (1995) reported an increase in the size of the mature somatic embryos and plantlet development of *Picea pungens* Engelm. in the culture medium supplemented with Asn. The combination of several amino acids (Gln, Asn, Arg, L-citrulline, L-ornithine, L-lysine, L-alanine, and Pro) was also beneficial in *C. japonica* SE, and the increase of concentration of several amino acids (2 gL^–1^ of Gln, 1 gL^–1^ of Asn, and 0.5 gL^–1^ of Arg) was essential to improve an efficient maturation of ses (>1,000 sesg^–1^ fresh weight) (Maruyama et al., 2021). In addition, the inclusion of a mixture of amino acid solution with 17 amino acids (Gln, alanine, Pro, lysine, glycine, Arg, leucine, phenylalanine, serine, isoleucine, valine, histidine, threonine, tyrosine, Asn, tryptophan, and cysteine) in the maturation medium of *P. patula* tended to increase the production of ses and the later conversion of ses in plantlets (Malabadi and Van Staden, 2005). *P. halepensis* ses developed in culture media with MIX III (2,200 mgL^–1^ of Gln, 525 mgL^–1^ of Asn, 175 mgL^–1^ of L-Arg, 17.5 mgL^–1^ of Pro, 19.75 mgL^–1^ of L-citrulline, 19 mgL^–1^ of L-ornithine, 13.75 mgL^–1^ of L-lysine, and 10 mgL^–1^ of L-alanine) were smaller and showed better germination rates (Figure 4C). These results are in agreement with those found in *Pinus strobus* L. where an increase in the concentration of Gln in the maturation medium for an embryogenic line resulted in an increase in the number of ses (Garin et al., 2000). Our findings show the important role of Gln in the maturation of *P. halepensis*, enhancing the germination of ses obtained from the maturation medium with a 4-fold increase in this amino acid concentration. This result coincides with some authors who report the use of Gln as one of the main sources of organic nitrogen for SE in *Plinia peruviana* Poir. Govaerts, *Pinus koraiensis* Siebold, and Zucc. and *Viola canescens* Wall. Ex. Roxb. (Silveira et al., 2020; Gao et al., 2021; Khajuria et al., 2021). In agreement with the improvements observed in the germination of *P. halepensis* ses, it was reported that the Gln also influenced the growth of pro-embryogenic masses in *Picea abies* L. Karst. (Carlsson et al., 2017) and *P. peruviana* (Silveira et al., 2020), increasing production of ses and consequent conversion to viable plants in date palm (El-Dawayati et al., 2018), as well as induction of secondary ses in *Quercus suber* L. (Rahmouni et al., 2020).

In relation to the carbohydrate source in a maturation medium, our results showed a different response in *P. radiata* and *P. halepensis.* In this sense, production, germination, and acclimatization of *P. radiata* ses were better in the presence of sucrose than in maltose in agreement with results obtained in *Pinus pinaster* Ait. (Ramarosandratana et al., 2001; Klimaszewska et al., 2007). Although, in our results, we did not see an improvement in the NNE at high carbohydrate concentrations in *P. radiata*, in *P. strobus*, the increase in its concentration (350 mM) produced a high amount of ses (Garin et al., 2000). Unlike *P. radiata*, the presence of 175 mM of maltose in *P. halepensis* maturation media promoted an increase in the size of ses, with a significant improvement in their germination rates. In this sense, plantlets obtained from *P. halepensis* ses developed in maturation media with 175 mM of maltose had an increase in the aerial part, and number of secondary roots and 100% of acclimatization was observed. Our results for *P. halepensis* are in agreement with those found in other *Pinus* species such as *P. patula* (Malabadi and Van Staden, 2005), *Pinus thunbergii* Parl. (Sun et al., 2021), and *Pinus elliottii* Engelm. (Yang et al., 2020).

Scott et al. (1995), studying the metabolism of maltose and sucrose in the embryogenesis of microspores isolated from *Hordeum vulgare* L., reported a higher assimilation of carbon in the culture medium, containing sucrose than in those containing maltose, probably because sucrose is well assimilated in both gymnosperms and angiosperms (Ameri et al., 2020; De Sousa et al., 2020; Peng et al., 2020; Shirin et al., 2020). However, different authors have shown controversial results; in this sense, Scott et al. (1995) associated the induction of embryogenesis with lower rates of substrate accumulation obtained from culture media containing maltose, and Carrion Pereira et al. (2019) described maltose as more efficient than sucrose to regenerate *Urochloa brizantha* cv. “Marandu” plants. Even when there are no reports describing morphological characteristics of ses with the concentration of maltose besides ours for *P. halepensis*, an increase in the production of the number of ses in *Pinus massoniana* Lamb. and *P. elliottii* has been reported (Yang et al., 2020; Xia et al., 2021). Similar to our results for *P. halepensis*, Salaj et al. (2019) reported that the presence of maltose in the maturation medium was essential for the formation ses with a consequent conversion of *P. nigra* plantlets.

In addition to morphological characteristics, the changes in the maturation medium also affected the PAs contents in germinated ses. PAs are extremely important for plants and are related to abiotic and biotic stress tolerance (Zou et al., 2021). Also, the Pas vary between species and the developmental stages of SE (Minocha et al., 1999; Silveira et al., 2004; Kuznetsov and Shevyakova, 2007; de Oliveira et al., 2020; Botini et al., 2021). In our work, the amount of PAs did not change with different treatments assayed in *P. radiata*. On the contrary, we observed that the amount of Put was more affected by the different treatments during the maturation stage than other Pas in *P. halepensis*. Moreover, this is the first report about the presence of endogenous Cad in *Pinus* spp. In this sense, concentrations of Cad were observed in the germinated ses obtained in both species and regardless of the treatments assayed. Cad is a lysine decomposition product, rare in plants, and associated with the accumulation of other Pas (Liu et al., 2014; Jancewicz et al., 2016). In our studies, the presence of Cad could not be associated with worse acclimatization, although, in *Pinus sylvestris* L., the root formation decreased in the presence of exogenous Cad (Niemi et al., 2002). In *Arabidopsis thaliana* L. Heynh., the presence of exogenous Cad was associated with the alterations in the morphological characteristics that modulate plant development, as well as environmental stress responses (Liu et al., 2014). In addition, in the same species, Cad inhibited primary root growth, and it was related to skewing, waving, and lateral root formation (Strohm et al., 2015). Moreover, it is possible to study the relationship of the Cad content with future stress experiments in *Pinus*, knowing that the Cad can improve the tolerance of abiotic stress in several species with an overexpression of the genes responsible for stress tolerance (Rajpal and Tomar, 2020).

Furthermore, differences between PAs contents were reported as important in SE. The reduction in Put content and the increase in other PAs (Spd, Spm, and Cad) during SE enabled the correct development of ses; for this reason, those differences have been used as important biochemical parameters for the selection of cell lines with embryogenic potential in *A. angustifolia* and *P. radiata* (Minocha et al., 1999; Jo et al., 2014; de Oliveira et al., 2015). In this work, regardless of the treatment of amino acids mixture, we observed an increase of Cad in relation to Put and Spm, but not in relation to Spd in the germinated *P. radiata* ses. Also, levels of Put were the lowest of all PAs detected in germinated ses; a similar profile was reported by Mikula et al. (2021) in *Cyathea delgadii* Sternb. SE process in which profiles of Cad and Spd increased in relation to other PAs. In agreement with these results and in opposition to ours, Minocha et al. (1999) reported that the most abundant PAs during the development of zygotic and ses in *P. radiata* was Spd in relation to Put and Spm. In contrast to our results, in the maturation of *Picea glauca* (Moench) Voss, the amount of Put was higher than Spd (Kong et al., 1998).

In *P. halepensis*, in MIX I, there were higher amounts of Cad, Spd, and Spm than in the remaining combinations, although there were no significant differences. In this sense, *Vicia faba* L. was reported that foliar application with different concentrations of amino acids mixture [Arg (5.2–6.2%), Pro (2.23–3.5%), Tiroanine (3.05–3.56%), Aspartic acid (3.2–3.45%), Serine (3.76–4.49%), Glutamic acid (7.24–9.12%), Lysine (1.87–2.45%), Alanine (2.16–2.20%), Cysteine (1.87–2.45%), Valine (2.8–3.1%), Methionine (0.23–0.3%), Isoleucine (1.26–1.7%), Leucine (1.98–2.8%), Tyrosine (0.48–1.02%), Phenylalanine (1.03–1.78%), lysine (1.39–2.3%), and Histidine (0.42–0.9%)] improved the PAs contents in the plants grown under seawater salinity stress (Sadak and Abdelhamid, 2015). Similar levels of Put and the other PAs were observed in germinated ses of *P. halepensis* from a maturation medium with an increase of 350 mgL^–1^ of L-Arg content (MIX II). However, with 2,200 mgL^–1^ of Gln (MIX III) in the maturation medium, a decrease in Put was observed in the germinated ses for *P. halepensis*. Our results are in agreement with those reported by Nieves et al. (2008), which showed that the addition of Arg in the culture medium of *Saccharum* spp. is related to the changes in the amount of PAs, especially Put. The observed levels of Put in our results can be explained due to PAs derived from amino acids through decarboxylation, and the Put can be synthesized from the arginine (Bagni and Tassoni, 2001; de Oliveira et al., 2015). Furthermore, the Put is a direct substrate for Spd and Spm (Mustafavi et al., 2018). For example, in embryogenic masses of *P. sylvestris*, the highest detected PA was Put compared to Spd and Spm (Salo et al., 2016). High amounts of Put appeared both in embryogenic lines that produced ses and in those that did not produce ses, while Spd was directly associated with the embryogenic lines and was able to produce ses in *P. sylvestris* (Salo et al., 2016). In *P. nigra*, the increase in the total amounts of free PAs was related with a lower embryogenic potential (Noceda et al., 2009). Meanwhile, the Spm was crucial to regulate the stress responses in *A. thaliana*, *Rosa damascene* Mill., and *Citrus reticulata* L., among others (Yamaguchi et al., 2007; Shi et al., 2010; Hassan et al., 2018; Hasan et al., 2021).

## Conclusion

An improvement in the germination of ses, conversion of ses in plantlets, and the acclimatization of plantlets were obtained in *P. radiata* and *P. halepensis* with alterations in the amino acid mixture or carbohydrate sources/concentrations in the maturation medium. Furthermore, changes in the maturation medium were not affected by the amount of PA levels in the ses of *P. radiata* but changed in *P. halepensis*. Cadaverine was detected in germinated ses of *P. radiata* and *P. halepensis*.

## Data Availability Statement

The raw data supporting the conclusions of this article will be made available by the authors, without undue reservation.

## Author Contributions

PM, IM, and AN conceived and planned the experiments. AN performed the experiments and wrote the manuscript. AC-O and AN carried out the statistical analyses for the amino acid supplementation experiment, and the carbohydrate supplementation experiment, and the characterization of the plantlets. AN, LP, FB, MG, and NS carried out the polyamines analysis. All authors provided critical feedback and helped shape the manuscript.

## Conflict of Interest

The authors declare that the research was conducted in the absence of any commercial or financial relationships that could be construed as a potential conflict of interest.

## Publisher’s Note

All claims expressed in this article are solely those of the authors and do not necessarily represent those of their affiliated organizations, or those of the publisher, the editors and the reviewers. Any product that may be evaluated in this article, or claim that may be made by its manufacturer, is not guaranteed or endorsed by the publisher.
